# Elevated cardiac troponin in the early post-operative period and mortality following ruptured abdominal aortic aneurysm: a retrospective population-based cohort study

**DOI:** 10.1186/cc11461

**Published:** 2012-08-07

**Authors:** Ilana Kopolovic, Kimberley Simmonds, Shelley Duggan, Mark Ewanchuk, Daniel E Stollery, Sean M Bagshaw

**Affiliations:** 1 University of Alberta Hospital, Division of Critical Care Medicine, Faculty of Medicine and Dentistry, University of Alberta, 3C1.12 Walter Mackenzie Centre, 8440 - 112 Street, Edmonton, AB T6G 2B7, Canada; 2 Infectious Disease Epidemiology, Surveillance and Assessment Branch, Community and Population Health Division, Alberta Health & Wellness, 23rd Floor, Telus Plaza NT, 10025 Jasper Avenue, Edmonton, AB T5J 1S6, Canada; 3 Grey Nuns Community Hospital, Division of Critical Care Medicine, Faculty of Medicine and Dentistry, University of Alberta, 100 Youville Dr W, Edmonton, AB T6L 5X8, Canada

## Abstract

**Introduction:**

Cardiac complications are potentially life-threatening following emergency repair of ruptured abdominal aortic aneurysms (rAAA). Our objectives were to describe the incidence, risk factors, cardiac outcomes and mortality associated with elevated cardiac-specific troponin (cTnI) following repair of rAAA. We hypothesized that early post-operative cTnI elevation (>0.15 mcg/L) in rAAA patients would identify a high-risk subgroup for cardiovascular complications and adverse outcomes.

**Methods:**

This was a retrospective population-based cohort study of all referrals for emergency repair of rAAA in central and northern Alberta, from 1 January 2002 to 31 December 2009. Demographic, clinical, physiologic and laboratory data were extracted, along with cardiac-specific investigations and events in the 72 hours following rAAA repair.

**Results:**

In total, 55% of patients (*n *= 77/141) had elevated cTnI, of which 12% (*n *= 9) had ST segment elevation, 23% (*n *= 18) had ST segment depression, 5% (*n *= 4) had other ECG changes, and 61% (*n *= 47) had no diagnostic ECG changes. Those with positive cTnI were more likely to have coronary artery disease (45.5% vs. 23.4%, *P *= 0.01) and higher Acute Physiology and Chronic Health Evaluation (APACHE) II scores (24.9 vs. 21.4, *n *= 0.016). cTnI positive patients were more likely to receive vasoactive support (58.4% vs. 14.1%, *P *< 0.001), had longer intensive care unit (ICU) lengths of stay (8 (3 to 11) vs. 4 (2 to 9) days, *P *= 0.02) and higher adjusted in-hospital mortality (40.3% vs. 14.1%; OR 4.23; 95% CI, 1.47 to 12.1; *P *= 0.007).

**Conclusions:**

Elevated cTnI early after rAAA repair is an independent predictor for post-operative complications and death.

## Introduction

Rupture of an abdominal aortic aneurysm (rAAA) is a life-threatening condition requiring emergency repair. Open vascular surgical repair, when coupled with the high prevalence of co-morbid illness in these patients, in particular cardiovascular disease, can translate into significant risk for post-operative complications and morbidity [1].

The development of peri-operative myocardial injury has prognostic importance for survivors of emergency rAAA repair. In a small retrospective study of 101 high-risk rAAA patients surviving to receive operative repair, 31% of early post-operative deaths were attributed to acute myocardial infarction (AMI) [2]. In addition, a small retrospective study found that coronary artery disease (CAD) was the most common late cause of death in survivors of rAAA repair [3]. These observations highlight that patients with rAAA have a high prevalence of CAD, which may be subclinical. These patients are susceptible to myocardial injury, which is associated with increased morbidity and mortality.

There are numerous biomarkers available for use in critical illness with the aim of providing incremental diagnostic and prognostic information and to better inform decision-making. However, as recently shown in a review of biomarkers in sepsis, of the 178 biomarkers identified, many lacked sufficient sensitivity and specificity to be reliably applied in clinical practice [4]. On the other hand, cardiac-specific troponins are highly unique to cardiac muscle. Troponin-I (cTnI) and troponin-T (cTnT) are integral regulatory proteins involved in actin-myosin interaction to facilitate myocardial contraction [5]. Accordingly, this has provided an opportunity to develop highly sensitive and specific quantitative assays for myocardial necrosis through early detection of elevated plasma troponin in patients suspected of having myocardial injury (both approaching 90 to 100% by 6 to 12 hours) [6]. Importantly, while these troponin assays remain highly sensitive and specific for myocardial necrosis, they are unable to discriminate the precise mechanism of myocardial injury [7].

Recently, the VISION study (Vascular Events in Non-cardiac Surgery Patients Cohort Evaluation) reported an 11.6% incidence of a high-sensitivity cTnT (fourth generation) ≥0.02 ng/mL in 11.6% of patients aged 45 years or older within three days of undergoing non-cardiac surgery. While corresponding electrocardiogram (ECG) changes suggestive of myocardial ischemia were not reported, elevated cTnT showed strong independent association with higher 30-day mortality. Notably; however, very few patients included in VISION received emergency major vascular surgery [8]. Two small cohort studies, both focused on elective AAA repair, found early post-operative elevations in cardiac-specific cTnI in 46 and 58%, respectively [9,10]. Most studies to date have generally been limited in scope, have evaluated only elective AAA repair and have often focused only describing biochemical myocardial injury rather than correlating these data with a post-operative course, cardiovascular events and outcomes.

We hypothesized that elevated cTnI in the early post-operative period would be predictive of a more complicated course and less favorable outcome. Our objectives were: (a) to describe the incidence and pattern of cTnI elevation after repair of rAAA; (b) to describe the occurrence of electrocardiographic (ECG) and echocardiographic (ECHO) evidence of myocardial ischemia and/or dysfunction after repair of rAAA; (c) to describe the post-operative clinical course, treatment intensity and occurrence of cardiac complications after repair of rAAA; and (d) to describe the short- and long-term mortality associated with elevated cTnI after repair of rAAA.

## Materials and methods

The study protocol was approved by the Health Research Ethics Board at the University of Alberta prior to commencement. The requirement for written consent was waived. The reporting of this study follows the STROBE guideline [11].

### Design, setting and participants

This was a retrospective population-based cohort study. The study was performed in the province of Alberta, Canada (population approximately 3.7 million in 2009). In Alberta, there are eight geographical health zones and two regional referral centers for all major vascular surgery. In the five health zones comprising central and northern Alberta (population approximately 2.0 million), all major vascular surgery and all cases of rAAA surviving to hospital are referred to the Grey Nuns Community Hospital (GNH). The GNH is a university-affiliated, 347-bed hospital located in Edmonton, Alberta. The GNH intensive care unit (ICU) is a closed, eight-bed mixed medical-surgical unit staffed by dedicated intensivists and receives approximately 500 admissions annually. All patients with emergency repair of rAAA are monitored and supported in the ICU post-operatively. All adult (≥18 years) patients admitted to the GNH with a primary diagnosis of rAAA undergoing surgical repair during the study period were included.

### Study protocol

Patients were identified by query of an ICU-specific clinical and/or administrative database (Minimal Data Set (MDS) database) between 1 January 2002 and 31 December 2009. The MDS database is maintained by the regional Division of Critical Care Medicine, and routinely captures demographic, clinical, physiologic and outcome data on all admissions to the five participating intensive care units in Edmonton. We extracted data on patient demographics, ICU admission source, ICU admission time, post-operative status, co-morbid conditions, primary ICU admission diagnoses, necessity for mechanical ventilation, vasoactive support, Acute Physiology and Chronic Health Evaluation (APACHE) II score, ICU and hospital duration of stay, and ICU, in-hospital and one-year mortality. Individual patient medical records were also interrogated for additional laboratory investigation data (that is, cTnI), details of in-hospital consultations, peri-operative complications (that is, myocardial infarction, cardiogenic shock) and operative/anesthesia records (that is, procedure duration, blood loss, and transfusions) and discharge summaries.

All patients had routine serial measurement of serum cardiac-specific troponin I (cTnI) for a minimum of 72 hours post-operatively. cTnI was measured using the Beckman-Coulter assay which detects the presence of cTnI at a threshold concentration of 0.15 mcg/L [12]. Any cTnI ≥0.15 mcg/L was designated a positive result (TnI+). Daily ECGs were evaluated for patterns of myocardial injury. Any bedside echocardiography (ECHO) reports were reviewed to provide data on left ventricular hypertrophy (LVH), systolic function/left ventricular ejection fraction (LVEF), diastolic dysfunction and regional wall motion abnormalities.

### Operational definitions

Patients were said to have a history of coronary artery disease (CAD) if this was documented in their medical records. Presence of diabetes mellitus (DM) was defined by prior documentation in the medical record or by an admission HbA1c ≥6.5%. Hypertension was diagnosed if documented in the medical record. ECG-ST segment elevation and depression were defined as a new deviation of ≥1 mm from baseline in limb ECG leads and ≥2 mm in precordial ECG leads. Congestive heart failure (CHF) was defined as hypoxemia and radiographic evidence of pulmonary edema attributed to impaired cardiac function and/or documentation by the intensivist/cardiologist of CHF based on clinical evaluation. Cardiogenic shock was defined as systemic hypotension and/or end organ hypo-perfusion attributed to inadequate cardiac output after volume resuscitation and/or the need for inotropic therapy.

### Statistical analysis

The primary exposure of interest was elevation in cTnI in the first 72 hours post-operatively. The primary outcome measure was in-hospital mortality. The secondary outcome measures were ICU and one-year mortality, ICU and hospital lengths of stay and occurrence of cardiovascular complications. We analyzed univariate associations between patient variables and outcomes, stratified by cTnI status (positive or negative). Continuous normally or near normally distributed data are reported as means with standard deviations (SD) and compared by Student's *t*-test. Non-normally distributed continuous data are reported as medians with inter-quartile ranges (IQR) and were compared by Mann Whitney U-test. Categorical variables were compared using the two-sided Fisher exact test or the Chi-squared test. A customized, multiple variable logistic regression model was created, using in-hospital mortality as the dependent variable, that considered age, pre-existing coronary artery disease, APACHE II score, intra-operative blood loss, vasopressor use, post-operative acute kidney injury (AKI), ECG changes and cTnI as covariates. ECG changes were omitted from the final model due to significant correlation with cTnI. Data are reported as odds ratios (OR) with 95% confidence intervals (CI). Model calibration and fit were assessed by the area under the receiver operating characteristic curve (AUC) and the Hosmer-Lemeshow goodness of fit (GoF) test, respectively. Crude survival stratified by AKI was assessed graphically by the Kaplan-Meier product limit estimator and compared with the log-rank test. All statistical analyses were two-sided and *P *< 0.05 was considered significant. Statistical analyses were conducted using Intercooled Stata Release 11.2 (Stata Corp., College Station, TX, USA).

## Results

In total, 141 patients were admitted to the ICU following rAAA repair. The estimated median (IQR) distance from the patient's residence to hospital was 34 km (18 to 149). Mean (SD) age was 71 (9) years, 85.8% (*n *= 121) were male, 79.4% (*n *= 112) had a smoking history; 35.5% (*n *= 50) had documented CAD and 20.0% (*n *= 28) had prior myocardial infarction (Table 1).

**Table 1:** Summary of baseline characteristics for rAAA patients stratified by cTnI status

	All patients (*n *= 141)	cTnI(+) (*n *= 77)	cTnI(-) (*n *= 64)	*P*
**Age (mean (SD))**	71(9)	72 (8)	71 (10)	0.74
**Male (n, %) **	121 (85.8)	67 (87.0)	54 (84.4)	0.83

**Co-morbidities**				

**Coronary artery disease (n, %)**	50 (35.5)	35 (45.5)	15 (23.4)	0.008
**Diabetes mellitus (n, %)**	24 (17.0)	17 (22.1)	7 (10.9)	0.12
**Previous myocardial infarction (n, %)**	28 (20.0)	19 (24.7)	9 (14.1)	0.17
**Hypertension (n, %)**	99 (70.2)	60 (77.9)	39 (60.9)	0.04
**Current smoker (n, %)**	67 (47.5)	33 (44.0)	34 (53.1)	0.30
**Ever smoked (n, %)**	112 (79.4)	58 (75.3)	54 (84.4)	0.26

**Medications (prior to admission)**				

**Beta-blocker (n, %)**	39 (27.7)	28 (36.4)	11 (17.2)	0.01
**Statin (n, %)**	42 (29.8)	28 (36.4)	14 (21.9)	0.09
**ACE I/ARB (n, %)**	46 (32.6)	29 (37.7)	17 (26.6)	0.22

**Peri-operative variables**				

**Heart rate (peak) (BPM) (mean (SD))**	110 (20)	113 (19)	107 (21)	0.08
**Atrial fibrillation (n, %)**	22 (15.6)	19 (25.0)	3 (4.6)	0.001
**APACHE II score (mean (SD))**	23.3 (8.4)	24.9 (8.6)	21.4 (8.0)	0.02
**Operative time (minute) (mean (SD))**	230 (75)	231 (75)	228 (77)	0.82
**EBL (L) (mean (SD))**	3.4 (3.3)	3.6 (3.0)	2.9 (3.6)	0.21
**Hgb (g/L) (nadar) (mean (SD))**	83.6 (16.0)	81.6 (15.2)	86.0 (16.7)	0.10
**Hgb (g/L) (delta) (mean (SD))**	54.4 (22.9)	57.0 (21.4)	50.8 (24.5)	0.12
**Vasoactive therapy (n, %)**	54 (38.3)	45 (58.4)	9 (14.0)	<0.001
**ECG Changes (n, %)**	32 (22.7)	32 (43.0)	0 (0)	-

Abbreviations: ACE, angiotensin converting enzyme; APACHE, acute physiology and chronic health evaluation; ARB, angiotensin receptor blocker; bpm, beats per min; EBL, estimated blood loss; Hgb, hemoglobin

### Clinical characteristics of cTnI+ patients

In total, 55% (*n *= 77) of patients had an elevated cTnI (cTnI+) in the first 72 hours post-operatively, whereas 45% (*n *= 64) had no elevation in cTnI (cTnI-) (Table 1). cTnI+ patients were more likely to have pre-existing CAD (45.4% vs. 23.4%; OR 2.72; 95% CI, 1.31 to 5.62, *P *= 0.008), hypertension (77.9% vs. 60.9%; OR 2.26; 95% CI, 1.09 to 4.69, *P *= 0.04) and to be prescribed beta-blockers prior to hospital admission (36.3% vs. 17.2%; OR 2.75; 95% CI, 1.25 to 6.04, *P *= 0.01). Forty-three percent (*n *= 32) of cTnI+ patients had acute ECG changes consistent with acute ischemia, compared with none of the cTnI- patients.

Of those cTnI+ patients, the initial rise occurred within the 24 hours of operative repair in 59.7% (*n *= 46), whereas 36.4% (*n *= 28) occurred in the subsequent 24 hours (between 24 and 48 hours), and the remaining 5.2% (*n *= 4) occurred >48 hours following surgery. In cTnI+ patients, the median (IQR) peak value of cTnI was 1.43 mcg/L (0.53 to 3.04). Peak elevation in cTnI occurred on the first, second or on or after the third post-operative day in 39.0%, 35.1%, and 26.0%, respectively.

### Clinical course

cTnI+ patients had higher APACHE II scores (*P *= 0.02) and were more likely to have received post-operative vasoactive support (58.4% vs. 9.3%; OR 13.6; 95% CI 5.3 to 34.4, *P *< 0.001). There was a trend for cTnI+ patients to have lower nadir and greater relative change in intra-operative hemoglobin compared with cTnI- patients (Table 1). Post-operative CHF was significantly more common in cTnI+ patients (41.6% vs. 14.0%; OR 4.35; 95% CI 1.90 to 9.90, *P *< 0.001) compared with cTnI- patients (Table 2). Likewise, the occurrence of cardiogenic shock was more likely in cTnI+ patients (26.0% vs. 1.6%; OR 22.1; 95% CI 3.63 to ∞, *P *< 0.001) compared with cTnI- patients.

**Table 2:** Summary of complications and clinical outcomes for rAAA patients stratified by cTnI status

Outcome	All patients (*n *= 141)	cTnI (+) (*n *= 77)	cTnI (-) (*n *= 64)	*P*
**Congestive heart failure (n, %)**	41 (29.1)	32 (41.6)	9 (14.0)	<0.001
**Cardiogenic shock (n, %)**	21 (14.9)	20 (26.0)	1 (1.6%)	<0.001
**Acute kidney injury (n, %)**	98 (71.0)	61 (82.4)	37 (57.8)	0.002
**Renal replacement therapy (n, %)**	18 (13.3)	15 (20.6)	3 (4.8)	0.01
**LOS (d) hospital (med (IQR))**	12 (8 to 20)	12 (7 to 20)	11 (9 to 19)	0.84
LOS hospital (d) (survivors)* (med (IQR))	13 (10 to 22)	16 (11 to 24)	12 (9 to 17)	0.02
**LOS ICU (d) (med (IQR))**	5 (2 to 11)	8 (3 to 13)	4 (2 to 9)	0.03
LOS ICU (d) (survivors)* (med (IQR))	7 (3 to 11)	8 (4 to 15)	4 (2 to 9)	0.002
**ICU mortality (n, %)**	30 (21.4)	24 (31.6)	6 (9.4)	0.01
**In-hospital mortality (n, %)**	40 (28.3)	31 (40.3)	9 (14.1)	<0.001
**1-year mortality (n, %) (*n *= 139)**	48 (34.5)	35 (46.7)	13 (20.3)	0.001

*sub-analysis of survivors, total *n *= 101, troponin (+) *n *= 46, troponin (-) *n *= 55. Abbreviations: ICU, intensive care unit; LOS. length of stay.

### Mortality

**Figure 1:**
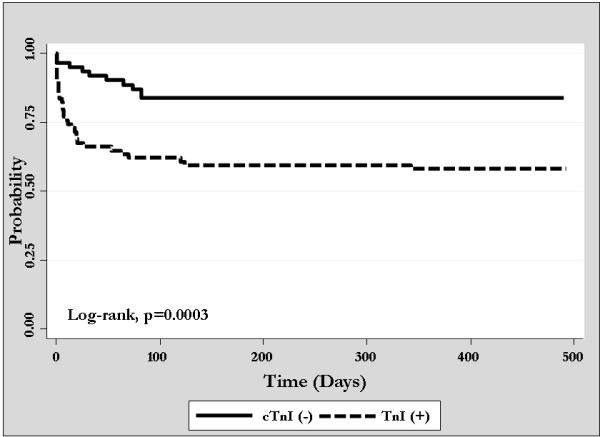
**Crude Kaplan-Meier survival stratified by post-operative cTnI status**.

Intensive care unit, in-hospital and one-year mortality rates were 21.4% (*n *= 30), 28.3% (*n *= 40) and 34.0% (*n *= 48), respectively. In-hospital mortality was significantly higher for cTnI+ as compared with cTnI- patients (40.3% vs. 14.1%; OR 4.11; 95% CI 1.80 to 9.38, *P *< 0.001) (Table 2). Likewise, ICU (31.6% vs. 9.4%; OR 4.46; 95% CI 1.73 to 11.4, *P *= 0.002) and one-year (45.4% vs. 20.3%; OR 3.27; 95% CI 1.54 to 6.90, *P *= 0.002) mortality rates were also higher for cTnI+ as compared with cTnI- patients (Table 2; Figure 1). By univariate analysis, several additional factors were associated with in-hospital mortality, including older age, higher APACHE II score, greater intra-operative blood loss, need for peri-operative vasoactive support, acute ECG changes and development of AKI, CHF or cardiogenic shock in the post-operative period (Table 3). After adjustment for age, illness severity and intra-operative blood loss in multi-variable analysis, elevated cTnI remained independently associated with higher in-hospital death (OR 4.23; 95% CI 1.47 to 12.1; *P *= 0.007; AUC 0.87; 95% CI 0.81 to 0.94, GoF, *P *= 0.79).

**Table 3:** Factors associated with in-hospital mortality

	All patients (*n *= 141)	Deceased in hospital (*P *= 40)	Survived to discharge (*P *= 101)	*P*
**Age (mean [SD])**	71 (9)	74 (7)	70 (9)	0.01
**Male (n, %) **	121 (85.8)	34 (85.0)	87 (86.1)	>0.999

**Co-morbidities**				

**Coronary artery disease (n, %)**	50 (35.5)	14 (35.0)	36 (35.6)	>0.999
**Diabetes mellitus (n, %)**	24 (17.0)	11 (27.5)	13 (12.9)	0.07
**Previous MI (n, %)**	28 (20.0)	8 (20.0)	20 (19.8)	>0.999
**Hypertension (n, %)**	99 (70.2)	31 (77.5)	68 (67.3)	0.32
**Current smoker (n, %)**	67 (47.5)	16 (40.0)	51 (50.5)	0.35
**Ever smoked (n, %)**	112 (79.4)	29 (72.5)	83 (82.2)	0.29

**Medications (prior to admission)**				

**Beta-blocker (n, %)**	39 (27.7)	11 (27.5)	28 (27.7)	>0.999
**Statin (n, %)**	42 (29.8)	14 (35.0)	28 (27.7)	0.51
**ACE I/ARB (n, %)**	46 (32.6)	17 (42.5)	29 (28.7)	0.17

**Peri-operative variables**				

**APACHE II score (mean (SD))**	23.3 (8.4)	30.4 (9.0)	20.5 (6.3)	<0.001
**Operative time (minute) (mean (SD))**	230 (75)	217 (77)	236 (75)	0.18
**EBL (L) (mean (SD))**	3.4 (3.3)	5.0 (5.1)	2.8 (2.1)	0.01
**Hgb (g/L) (nadar) (mean (SD))**	83.6 (16.0)	71.8 (16.4)	88.2 (13.4)	<0.001
**Hgb (g/L) (delta) (mean (SD))**	54.4 (22.9)	62.9 (27.4)	50.9 (19.9)	0.01
**Vasoactive therapy (n, %)**	54 (38.3)	31 (77.5)	23 (22.3)	<0.001
**cTnI (+) (n, %)**	77 (54.6)	31 (77.5)	46 (45.5)	<0.001
**Acute ECG changes (n, %) **	32 (22.7)	18 (45.0)	14 (13.9)	<0.001

**In-hospital events**				

**AKI (n, %)**	98 (71.0)	35 (92.1)	63 (63.0)	0.001
**Cardiogenic shock (n, %)**	21 (14.9)	14 (35.5)	7 (6.9)	<0.001
**CHF in hospital (n, %)**	41 (29.1)	19 (47.5)	22 (21.8)	0.006

Abbreviations: ACE, angiotensin converting enzyme; AKI, acute kidney injury; APACHE, acute physiology and chronic health evaluation; ARB, angiotensin receptor blocker; EBL, estimated blood loss; Hgb, hemoglobin

There was no association between timing of the first elevated or peak post-operative cTnI and hospital mortality (Tables 4 and 5). Likewise, there was no significant difference in median (IQR) peak cTnI between hospital survivors and non-survivors (1.21 (0.49, 3.68) mcg/L vs. 1.43 (0.96, 2.84) mcg/L, *P *= 0.37). In cTnI+ patients, pre-existing CAD was associated with a lower in-hospital mortality compared to those with no CAD (26.4% vs. 52.4%; OR 0.33; 95% CI 0.13 to 0.86, *P *= 0.03). cTnI+ patients surviving to discharge also had considerably longer ICU and hospital lengths of stay compared with cTnI- patients, respectively (Table 2).

**Table 4:** Mortality stratified by post-operative day on which the initial elevation in cTnI was detected

Post-op day first rise	Total (*n *= 77)	1 (*n *= 46)	2 (*n *= 25)	≥3 (*n *= 6)	*P*
**ICU mortality (n, %)**	24 (31.6)	14 (30.4)	9 (36.0)	1 (16.7)	0.74
**Hospital mortality (n, %)**	31 (40.3)	17 (36.9)	12 (48.0)	2 (33.3)	0.67
**1-year mortality (n, %)**	35 (46.7)	19 (42.2)	14 (58.3)	2 (33.3)	0.40

Abbreviations: ICU, intensive care unit.

**Table 5:** Mortality stratified by post-operative day on which peak cTnI value occurred

Post-op day peak	Total (*n *= 77)	1 (*n *= 30)	2 (*n *= 27)	≥3 (*n *= 20)	*P*
**ICU mortality (n, %)**	24 (31.6)	11 (36.7)	7 (25.9)	6 (30.0)	0.71
**Hospital mortality (n, %)**	31 (40.3)	12 (40.0)	10 (37.0)	9 (45.0)	0.92
**1-year mortality (n, %)**	35 (46.7)	14 (48.3)	11 (42.3)	10 (50.0)	0.88

Abbreviations: ICU, intensive care unit.

### Impact of ECG changes in cTnI+ patients

**Figure 2:**
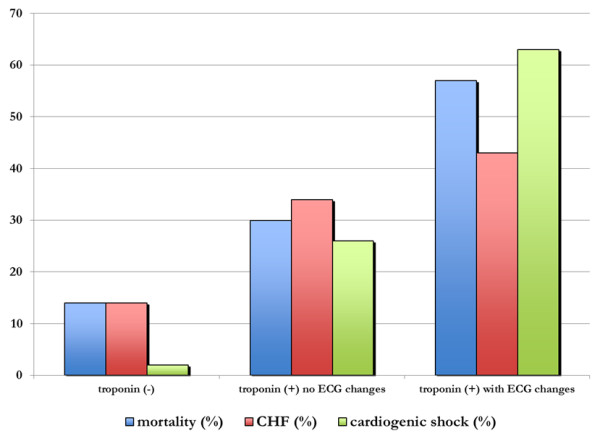
**Cardiac events and mortality stratified by post-operative cTnI status and ECG changes**. Groups differed significantly for mortality (*P *< 0.001), CHF (*P *= 0.004) and cardiogenic shock (*P *< 0.001).

ECG changes were present in 43.0% (*n *= 32) of cTnI+ patients. Of those with ECG changes, 28.1% (*n *= 9) had ST segment elevation; 56.3% (*n *= 18) had ST segment depression; and 12.5% (*n *= 4) had non-specific ST segment changes. ECG changes were a modifying factor for post-operative cardiac complications and mortality. The incidence of CHF, cardiogenic shock and in-hospital mortality were significantly higher in cTnI+ patients with ECG changes, compared to cTnI+ only and cTnI- patients without ECG changes (Figure 2). In addition, of cTnI+ patients, the presence of ECG changes were associated with a significantly higher odds of death compared to those with no ECG changes (56.3% vs. 29.3%; OR 3.11; 95% CI 1.19 to 8.11, *P *= 0.03).

### Post-operative investigations associated with cTnI+

In total, only 62.3% (*n *= 48) of cTnI+ patients were evaluated with ECHO. Of these, 54.1% (*n *= 26) were found to have an LVEF <60%, 45.8% (*n *= 22) had regional wall motion abnormalities, of whom eight had a prior history of prior myocardial infarction. ECHO evidence of reduced LVEF was associated with a higher likelihood of CHF, cardiogenic shock and mortality. Only one patient underwent multi-gated acquisition scanning (MIBI) after transfer from the ICU, which did not reveal any regional reversible ischemia. Only two patients received peri-operative cardiac catheterization; of which, only one received percutaneous coronary intervention (PCI), and both survived to hospital discharge.

## Discussion

We performed a retrospective population-based cohort study of patients undergoing emergency repair of ruptured AAA to describe the incidence, risk factors and outcomes associated with early post-operative elevation in cTnI.

### Summary of major findings

Elevation in cTnI in the 72 hours post-operative was common, occurring in 55% of patients, of which the majority occurred in the first 24 hours. We found that those patients with pre-existing CAD, hypertension or treatment with oral beta-blockers to be more likely to have elevated cTnI. We found cardiovascular complications, including CHF and cardiogenic shock, to be more likely in those with elevated cTnI. In addition, we found that elevated cTnI was independently associated with a higher risk for in-hospital mortality and longer stays in both ICU and hospital. Of interest, we found cTnI+ patients with documented pre-existing CAD had a lower observed in-hospital mortality compared to cTnI+ patients with no history of CAD. We found no association between timing of first or peak or absolute value of elevated cTnI and mortality. Among cTnI+ patients, only 43% had acute ECG changes; however, this subgroup had a higher risk of complications and mortality. Less than two-thirds of cTnI+ patients were investigated with echocardiography, but the majority of the echocardiograms performed demonstrated evidence of myocardial dysfunction and/or wall motion abnormalities. Finally, very few patients received further cardiac-specific investigations.

### Limitations

We recognize there are limitations with our study. First, it was a retrospective cohort study and, therefore, is prone to confounding and bias. Second, despite being a population-based surveillance over several years, our cohort remained relatively small, resulting in limited statistical power and susceptibility to errors of chance. Third, while post-operative cTnI and ECGs were routinely performed, there was no pre-specified protocol for further cardiac-specific investigations for cTnI+ patients and these were done at the discretion of the treating team. Fourth, we recognize our study spans several years, during which time revision to the cTnI assay may have been made. Furthermore, we recognize that newer fifth generation high-sensitivity assays with lower thresholds for detection of myocardial damage will soon be available for clinical use [5]. It is likely the greater utilization of these next generation assays will contribute to diagnostic and interpretive challenges for clinicians confronted with positive results in rAAA and other critically ill patients [13]. Finally, for patients undergoing in-hospital ECHO, we could not confirm whether prior ECHO had been performed, thus limiting our confidence in the acute nature of the ECHO findings.

### Comparison with prior literature

Prior studies evaluating the prognostic value of elevated post-operative cTnI in major vascular surgery have primarily focused on elective surgery [14]. Three prospective cohort studies have suggested post-operative cTnI elevation confers increased risk for complications [14-17]. Kim *et al. *found a 12% incidence of cTnI elevation (>1.5 mcg/L) within 72 hours of surgery. This was associated with an increased risk for AMI and dose-response increase in six-month mortality [15]. Landerberg *et al. *found 8.7% had a post-operative cTnI >1.5 mcg/L that likewise portended an increased risk for AMI and death. However, consistent with our data, the incidence of post-operative cTnI elevation in emergency vascular procedures has been shown to be considerably higher [18]. In an observational study of 50 surviving patients of emergency rAAA repair, Tambyraja *et al. *reported 46% had post-operative cTnI elevation; and similar to our study, they found an association with increased mortality and lengths of stay [10].

Biochemical evidence of myocardial injury (that is, elevated cTnI) should ideally be interpreted in the context of acute ECG changes consistent with an acute cardiac event. We found only 43% of our cTnI+ patients had acute ECG changes. Similarly, Tambyraja *et al. *reported 47.8% (*n *= 11/23) of cTnI+ patients had early post-operative acute ECG changes [10]. These data clearly confirm peri-operative cTnI evaluation coupled with acute ECG evidence of myocardial injury is associated with higher risk for complications and death. However, we have also shown that peri-operative cTnI "leak" not associated with acute ECG changes portends greater risk of complications and death that exceeds those with no cTnI elevation. This finding is supported by a recent meta-analysis of nine studies showing "isolated cTnI rise" was associated with increased 30-day mortality; however, the majority of studies included failed to specifically describe the occurrence of acute ECG changes [19]. Similarly, in a cohort of 211 high-risk patients undergoing emergency non-vascular surgery, Oscarrson *et al. *found elevated post-operative cTnI, in the absence of acute ECG changes, was predictive of major adverse cardiac events and 30-day mortality [18]. The majority of prevailing literature does not specifically describe the presence of acute ECG changes in association with elevated cTnI, or how these ECG findings potentially modify the post-operative risk of less favorable outcome. We believe our data extend these findings by suggesting incremental risk for cTnI elevation coupled with acute ECG changes over isolated cTnI elevation alone in the early post-operative period.

Our study confirmed the high prevalence of echocardiographic evidence of myocardial dysfunction and/or wall motion abnormalities in cTnI+ patients. These ECHO changes are also associated with an increased risk of complications and mortality. However, perhaps more importantly, given the increased peri-operative risk conferred by elevated cTnI, less than two in three patients were evaluated with post-operative ECHO; and fewer still underwent further cardiac-specific risk stratification. One major limitation of many prior studies of cTnI in vascular surgery has been the lack of assessment of myocardial function and/or description of cardiac-specific investigations [10,16,20]. We contend the finding of myocardial dysfunction and/or wall motion abnormalities in association with cTnI may represent true type I peri-operative myocardial infarction that under ideal circumstances would prompt early cardiac risk stratification and/or cardiac angiography. Likewise, elevated cTnI after repair of rAAA may also represent a failed cardiac "stress test" and serve to unmask previously undiagnosed and/or subclinical CAD. We also believe our data suggest these patients are at increased risk for cardiac morbidity and should receive further cardiovascular risk stratification.

Prior studies have not specifically described the post-operative clinical course (that is, hemodynamic compromise, congestive heart failure) associated with cTnI elevation [10,14-16]. Similar to prior studies [10], we found an association between cTnI+ and longer ICU and hospital stays. However, we were also able to show that cTnI+ patients suffered more complications (that is, congestive heart failure; cardiogenic shock; acute kidney injury) and also received greater treatment intensity (that is, vasoactive support) compared with cTnI- patients.

The risk associated with pre-operative beta-blocker use is of interest. This is clearly a complex issue and may not be modifiable in patients who have already taken their medications at the time of rupture and acute presentation to medical attention. Whether this associated risk represented a direct impact of the therapy or acute peri-operative withdrawal of therapy or whether beta-blockade was simply a marker of pre-existing CAD is unclear. Peri-operative beta-blockade does not benefit all patients undergoing non-cardiac surgery and has been associated with increased risk for complications and mortality [21,22]. Marston *et al. *described (non-significantly) that a higher proportion of cTnI+ compared with cTnI- patients were prescribed pre-operative beta-blocker therapy (88.6% vs. 77.2%, *P *= 0.18). In the POBBLE trial, peri-operative allocation to metoprolol during major elective vascular surgery resulted in a significantly greater utilization of intraoperative inotropic support; however, occurrence of major vascular events (approximately one-third) were not significantly different [20]. Instead, documentation of pre-operative beta-blocker should alert clinicians to the risk of increased susceptibility to myocardial injury and/or hemodynamic complications in the early post-operative period.

For those patients who survive repair of rAAA, the ideal peri-operative management strategies and/or interventions to mitigate the negative consequences associated with cTnI+ and optimize clinical outcome remains unknown. Our data not only confirm the high incidence of elevated cTnI+ associated with rAAA, but also the surprisingly high incidence of cardiac events, complications and the incremental risk of death. Importantly, our data also suggest that many of these patients receive either limited early investigations and/or suboptimal long-term cardiovascular risk stratification. Our findings are similar to the study by Kim *et al.*, in which all patients with post-operative acute myocardial infarction were managed medically, and none underwent coronary angiography within six months of the event [15]. In elective vascular surgical populations, in whom the negative prognostic value of isolated elevated cTnI has been more firmly established, ongoing clinical trials are evaluating early strategies for possible mitigation of long-term complications [23]. We contend additional research is needed with specific focus on the rAAA cohort. Patients with rAAA are unable to benefit from pre-operative modification of cardiovascular risk. Research is required to identify which peri-operative strategies might mitigate the significant complications associated with postoperative cTnI elevation in this setting.

## Conclusions

In summary, our population-based cohort study of patients undergoing emergency repair of ruptured AAA demonstrates a high incidence of early post-operative cTnI elevation. The cTnI+ cohort suffered a significant increase in cardiac events, complications and death. Optimal management of patients with cTnI elevation in this setting is unknown, and warrants further investigation to optimize outcomes in this group with significantly worse survival and poor prognosis.

## Key messages

• Elevated cTnI in the early post-operative period following rAAA repair was very common; however, only approximately one-third of patients showed acute ECG changes.

• Elevated cTnI occurred more commonly in patients with pre-existing coronary artery disease, hypertension and those prescribed pre-hospital beta-blocker therapy.

• Elevated cTnI was associated with greater risk of post-operative cardiovascular complications, including congestive heart failure and cardiogenic shock, along with higher resource use, and greater short-term mortality.

• Only two-thirds of patients were investigated with echocardiography in response to elevated cTnI; however, in those undergoing ECHO, findings of reduced ventricular function and wall motion abnormalities were common.

## Abbreviations

AKI: acute kidney injury; AMI: acute myocardial infarction; APACHE: Acute Physiology and Chronic Health Evaluation; AUC: area under the curve; CAD: coronary artery disease; CHF: congestive heart failure; CI: confidence interval; cTnI: cardiac-specific troponin I; cTnT: troponin-T; DM: diabetes mellitus; ECG: electrocardiography; ECHO: echocardiography; GNH: Grey Nuns Community Hospital; GoF: goodness of fit; ICU: intensive care unit; IQR: inter-quartile range; LVH: left ventricular hypertrophy; LVEF: left ventricular ejection fraction; MDS: Minimal Data Set; MIBI: Technetium (^99m^Tc) sestamibi nuclear imaging stress test; OR: odds ratio; PCI: Percutaneous coronary intervention; rAAA: ruptured abdominal aortic aneurysm.

## Competing interests

The authors declare that they have no competing interests.

## Authors' contributions

SMB and IK conceived of the study. SMB, DES, SD, ME and IK contributed to the study design, including formulating the research questions and ensuring a design to answer these. SMB, DES, SD and ME established access to data. KS and SMB conducted the statistical analysis. The manuscript was drafted by IK and SMB and its methods and intellectual content were reviewed by KS, DES, SD and ME, who each made revisions. All authors provided their approval of the final version of the manuscript.

## Authors' information

SMB is a critical care specialist attending general medical-surgical intensive care and cardiovascular surgical intensive care units at the University of Alberta Hospital, and an associate professor and clinical investigator at the University of Alberta. SD, DES and ME are critical care specialists attending the intensive care unit at the Grey Nuns Community Hospital. KS is an epidemiologist for Alberta Health Services. IK is a medical resident at the University of Alberta
